# A Vertebrate-Specific Chp-PAK-PIX Pathway Maintains E-Cadherin at Adherens Junctions during Zebrafish Epiboly

**DOI:** 10.1371/journal.pone.0010125

**Published:** 2010-04-12

**Authors:** Hwee Goon Tay, Yuen Wai Ng, Ed Manser

**Affiliations:** 1 RGS (Rho GTPases in Stem Cells) Group, Institute of Medical Biology (IMB), Singapore, Singapore; 2 sGSK (Small G-Protein Signaling and Kinases) Group, Institute of Molecular and Cell Biology (IMCB), Neuroscience Research Partnership, Singapore, Singapore; The Beatson Institute for Cancer Research, United Kingdom

## Abstract

**Background:**

In early vertebrate development, embryonic tissues modulate cell adhesiveness and acto-myosin contractility to correctly orchestrate the complex processes of gastrulation. E-cadherin (E-cadh) is the earliest expressed cadherin and is needed in the mesendodermal progenitors for efficient migration [1], [2]. Regulatory mechanisms involving directed E-cadh trafficking have been invoked downstream of Wnt11/5 signaling [3]. This non-canonical Wnt pathway regulates RhoA-ROK/DAAM1 to control the acto-myosin network. However, in this context nothing is known of the intracellular signals that participate in the correct localization of E-cadh, other than a need for Rab5c signaling [3].

**Methodology/Principal Findings:**

By studying loss of Chp induced by morpholino-oligonucleotide injection in zebrafish, we find that the vertebrate atypical Rho-GTPase Chp is essential for the proper disposition of cells in the early embryo. The underlying defect is not leading edge F-actin assembly (prominent in the cells of the envelope layer), but rather the failure to localize E-cadh and β-catenin at the adherens junctions. Loss of Chp results in delayed epiboly that can be rescued by mRNA co-injection, and phenocopies zebrafish *E-cadh* mutants [4], [5]. This new signaling pathway involves activation of an effector kinase PAK, and involvement of the adaptor PAK-interacting exchange factor PIX. Loss of signaling by any of the three components results in similar underlying defects, which is most prominent in the epithelial-like envelope layer.

**Conclusions/Significance:**

Our current study uncovers a developmental pathway involving Chp/PAK/PIX signaling, which helps co-ordinate E-cadh disposition to promote proper cell adhesiveness, and coordinate movements of the three major cell layers in epiboly. Our data shows that without Chp signaling, E-cadh shifts to intracellular vesicles rather than the adhesive contacts needed for directed cell movement. These events may mirror the requirement for PAK2 signaling essential for the proper formation of the blood-brain barrier [6], [7].

## Introduction

The ras-related Rho GTPases are known to play pivotal roles in a broad range of cytoskeletal activities that are required for cell migration, cell polarization and cytoskeletal rearrangements [8], [9], [10]. Several studies implicate Rho GTPases in cadherin-mediated cell-cell adhesion, which serves to coordinate cortical F-actin at these sites [11], [12], [13]. To date, there are 32 Rho genes identified in zebrafish which all have orthologues in the 23 gene products found in humans [14]. Rho pathways (exemplified by studies of the RhoA, Rac1 and Cdc42 proteins also present in invertebrates) participate in early embryonic development including gastrulation [15], [16], [17], [18], and neurulation [19]. Almost nothing is known regarding the ‘atypical’ Cdc42-like proteins which have arisen during vertebrate evolution [20].

During gastrulation, embryos undergo a series of morphogenetic events that simultaneously determine cell fates and the rearrangement of cells into three distinct germ layers. Early epiboly in zebrafish is the process that simultaneously allows blastodermal cells spread over the yolk cell, moving from the animal pole downwards to the vegetal pole [21], [22]. During the late blastula stage, the embryo then consists of an outer epithelium layer called the enveloping layer (EVL), the deep cell layer (DEL) and the yolk syncytial layer (YSL). The epithelial-like EVL is usually adhered to the YSL at its most vegetal margin, thereby sandwiches the DEL during epiboly. It takes about 10 hours post-fertilization (hpf) to completely cover the yolk at the end of gastrulation [21], [22]. In studies of *Fundulus*, the YSL has been shown to provide the driving force for these movements since it undergoes epiboly in the absence of the blastoderm [21]. Components of the cytoskeleton including F-actin and microtubules are required for epibolic movement [23], [24]. Not surprisingly, treatment of the actin toxin cytochalasin B causes failure to complete epiboly [21]. Microtubule disruption by either ultraviolet irradiation or nocodazole also impairs epiboly [25]. Gene mutations in zebrafish E-cadherin (E-cadh) including *half baked* and *cdh1^rk3^* exhibit defective epiboly [4], [5]. The *E-cadh* mutants cause arrest of deep cell layer movement but not the forward migration of EVL and YSL [4], [5]. Thus E-cadh- mediated cell-cell adhesion contributes to correct cell movement and rearrangement during epiboly. The underlying molecular mechanism of E-cadh regulation during epiboly remains to be elucidated.

In this study, we have used anti-sense morpholino-oligonucleotide (MO) to knock down Cdc42 and Chp (Cdc42 homologous protein), and found that the latter is essential for zebrafish epibolic morphogenesis. Chp/RhoV is one of a number of proteins related to the prototype yeast Cdc42; in zebrafish Cdc42-like GTPases; Cdc42a, Cdc42b, Cdc42c, RhoUa, RhoUb (Wrch1), RhoJ (TCL), and TC10 are reported [14]. Chp binds a number of effector kinases including PAKs [26], [27] and is reported as an early expressed neural crest marker in *Xenopus* [28]. Whole mount *in-situ* hybrization analysis indicates Chp and Wrch1 are expressed early in chick embryonic development, and during gastrointestinal tract development [29]. The Chp protein is interesting because unlike most GTPases, it is not C-terminal prenylated but rather undergoes C-terminal palmitoylation [30], which is a reversible modification. The Chp protein has been shown to promote rapid turnover of PAK1 when over-expressed in mammalian cells [27], however nothing is known of its biological role. We show here for the first time that Chp is required to stabilize the E-cadh/β-catenin complex at adherens junctions, and that in its absence, dynamic cell adhesion during epibolic movement is perturbed. The roles of the ubiquitous downstream components PAK and βPIX likewise are necessary to maintain E-cadh at the cell surface where we show that PAK activation at cell-cell junctions occurs downstream of Chp.

## Results

### The spatio- temporal expression of RhoV/Chp in zebrafish development

In a preliminary screen for Rho proteins essential for early vertebrate development, we found that a Chp anti-sense morpholino-oligonucleotide (MO), but not MOs directed to each of the three Cdc42 isoforms, led to defects in epiboly (see next section). The atypical Cdc42 homologue protein (Chp) was found in a screen for PAK2 partners by yeast two-hybrid screening [26], and can activate the JNK kinase pathway like other Rho proteins such as Rac1. The protein was subsequently termed RhoV in the zebrafish [14], chick [29] and frog [28], but we retain the original annotation. The biological functions of Chp include involvement in neural crest induction in *Xenopus* [28], with MO treatment resulting in a loss of cranial neural crest derived structures. Comparison of the primary amino acid sequence (Figure S1) of Chp from human and zebrafish indicates the effector regions (switch 1 and II) are and Rho inset domains well conserved. The zebrafish Chp has a shorter N-terminus suggesting this extension, and the presence of polyproline sequences, are not critical features of the GTPase. Chp has an unusual and well conserved carboxyl-terminus that terminates in CCFI and modified by palmitoylation [30]; this resembles the alternate spliced neuronal form of human Cdc42 [31] as shown in Figure S1.

**Figure 1:**
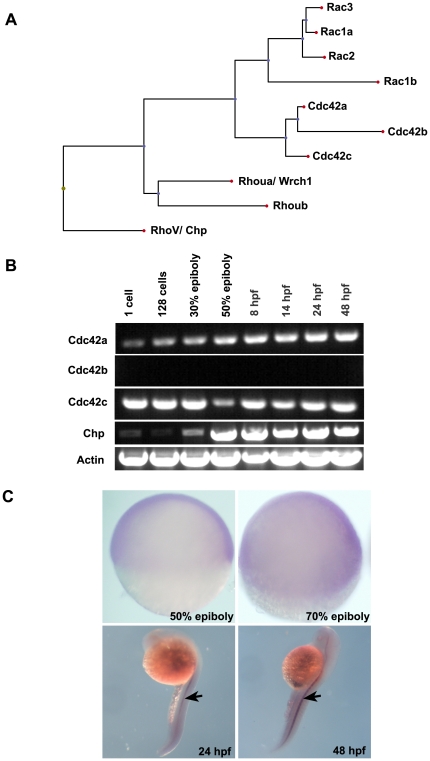
Protein sequence analysis and spatio-temporal expression of Chp. (**A**) The relationship between Chp, Cdc42 and Rac in zebrafish (*Danio rerio*). Protein sequences were aligned using ClustalW (DNAStar) and the dendrogram generated by Phylip. Accession numbers for cDNA sequences of *Cdc42 and Rac* family are: *Rac1a*, AY865568. *Rac1b*, XP_001332092.1. *Rac2*, AY865569. *Rac3*, AY865570. *Cdc42a*, AY865566. *Cdc42b*, XM_678979. *Cdc42c*, AY865567. *Rhoua*, AY865564. *Rhoub*, NM_001017784, Chp/ *RhoV*, NM_001012250. (**B**) RT-PCR products showing mRNA transcript profile of zebrafish Chp at different developmental stages as indicated. Both Cdc42a and Cdc42c were detected throughout the stages tested. Chp transcripts appear at epiboly. (**C**) Whole mount in situ hybridization (WISH) using dioxygenin (DIG) anti-sense Chp RNA probes indicates all cell types express the transcript at 50% and 80% epiboly (lateral views): Chp mRNA is enriched in notochord (indicated by arrows) at 24 hpf and 48 hpf.

Multiple alignment of these zebrafish Rho sequences [14] and their relatedness (Figure 1A) indicates Chp is the most divergent member of Cdc42 and Rac subfamily. We next examined the spatio-temporal expression of Chp by RT-PCR on embryos collected from zygote, blastula, gastrula, segmentation and pharyngula stages (Figure 1B). Chp transcripts accumulated significantly at 50% epiboly (shield stage) and were maintained up to 48 hpf. Of the three Cdc42 isoforms found in fish, two were expressed from the earliest stages (Figure 1B), and likely play redundant roles since no single MO directed to Cdc42 affected early development (data not shown). The spatial expression pattern of Chp was studied by whole-mount in situ hybridization (WISH) using an anti-sense rRNA probe. There was no obvious region-specific accumulation of mRNA at 50% or 80% epiboly (Figure 1C), but there was enriched signal in the notochord region at 24 and 48 hpf embryos.

### Reduced expression of Chp affects epibolic morphogenesis

**Figure 2:**
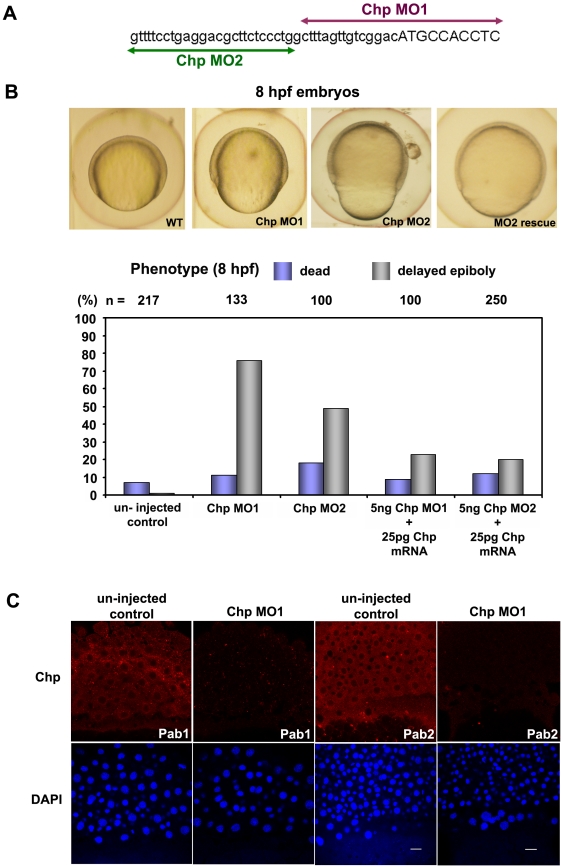
Chp function is required for epibolic movements. (**A**) Positions and sequence of Chp morpholino-oligoncleotides used in the study. The Chp MO1 and MO2 (corresponding to the reverse strand) are represented in purple and green respectively; lowercase sequence is 5′ UTR region. (**B**) Phenotypic examination of Chp morphants at 8 hpf revealed delayed closure of the yolk plug when compared with the un-injected controls. Rescue of the phenotype was performed by co-injecting Chp MO 1/2 with WT Chp (Zf) mRNA. Co-injection of 25 pg Chp mRNA with 5 ng Chp MO1/2 efficiently reduced the number of yolk plug defects at 8 hpf. Phenotypic analysis shows significant rescue in the delay of yolk plug closure at 8 hpf. (**C**) Immuno-histochemical staining of un-injected controls and Chp morphants at 60% epiboly using Chp antibody raised and purified from two animals (Pab1 and Pab2). Confocal images show endogenous Chp is strongly depleted Chp morphants. Decreased protein levels were observed in EVL, DEL and YSL layers with Chp MO. Embryos were counter-stained with DAPI. Scale bars = 20 µm.

To study the role of Rho proteins in the Cdc42 family, a number of MOs were designed and injected at the 1 cell stage. One of the MOs produced a clear defect in epiboly. This Chp MO1 targets sequences surrounding the initiation codon, and subsequently a Chp MO2 was designed against the Chp 5′ un-translated region (UTR) to confirm the phenotype (Figure 2A). In both cases, 5 ng MO gave similar epibolic phenotypes characterized by the presence of bulging yolk plug at the vegetal region and delayed epiboly (Figure 2B). This phenomenon was first observed at 60% epiboly, (cf. 8 hpf). Severe phenotype was characterized by a failure to close the yolk plug, and embryos did not survive to 24 hpf. This suggested that Chp is needed for epibolic morphogenesis where extensive cell movements drive the cells over the egg surface, while deep cells undergo gastrulation. Embryos injected with 25 pg of Chp at 1 cell stage were normal (data not shown). Resultant morphological defects in survivors of Chp MO1/2 at 24 hpf included shortened antero-posterior (A-P) axes, failure in yolk extension and rounded somites (data not shown). The specificities of the effects seen with Chp MOs were tested by co-injecting MO1 or MO2 synthetic mRNA encoding wild-type Chp (*Dr*). Examination of the embryos co-injected with 25 pg Chp mRNA indicated a significant rescue in the delay of yolk plug closure in both cases (Figure 2B). Thus Chp activity is essential for aspects of normal epibolic morphogenesis.

### MO treatment reduces Chp levels in all three major cell groups in epiboly

We considered that the role of Chp might involve events at the leading edge of the envelope layer (EVL) or yolk syncytial layer (YSL) cell margin during migration phase of epiboly. Antibodies raised against the synthetic peptide encoding the N-terminus of Chp (MPPQMDYFYHESRVP; affinity purified preparations Pab1 and Pab2) were tested for protein immuno-localization during epiboly. As for the RNA in-situ analysis, uniform Chp immuno-staining was also observed in the EVL, the deep layer (DEL) cells and in the YSL (Figure 2C). This staining was lost in the Chp morphants as anticipated. Epibolic morphogenesis in zebrafish requires the co-ordinated expansion of the EVL, DEL and YSL towards the vegetal pole [21], and it appeared likely that Chp plays a role in all three layers: this was borne out in our later studies. To our knowledge, this represents the first immuno-localization of Chp (previous studies involving only tagged GTPase).

### Chp MO does not affect mesodermal cell fate specification and early patterning

Chp knockdown was assessed for effects of early cell fate. WISH was performed to investigate the expression pattern of the mesodermal markers and organizer-specific genes; *chordin* (*Chd*), *Goosecoid* (*Gsc*), and *no tail* (*Ntl*) were tested at 50% epiboly or 70% epiboly (Figure S2). These markers are expressed in mesoderm precursor cells during early embryonic development. We conclude that Chp is not directly involved in mesodermal cell fate specification and early patterning, but disruption of Chp instead likely affects the final organization of these cells.

### Chp depletion does not disrupt the leading edge actin cytoskeleton

**Figure 3:**
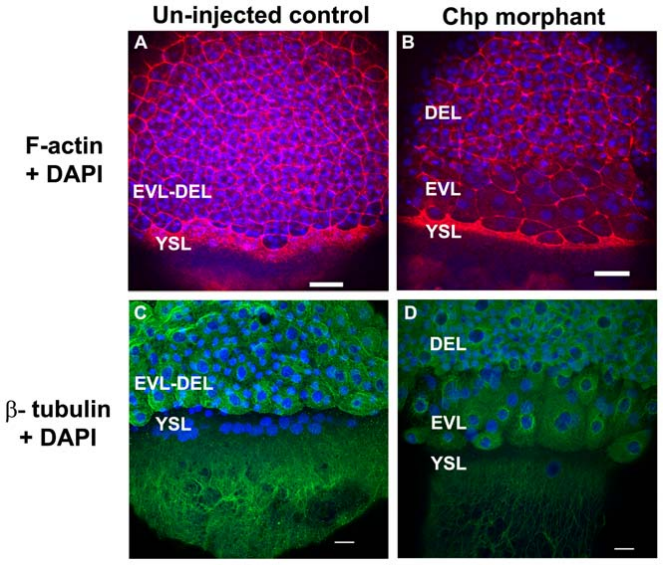
The actin cytoskeleton and microtubule networks in Chp morphants. Low resolution confocal images (10× objective) comparing (**A**) un-injected control and (**B**) Chp morphant with respect to F-actin organization at the vegetal margins of EVL and DEL, and at the external yolk syncytial layer (YSL). The deep cells marked by DAPI stained nuclei, fail to properly migrate in Chp morphants (**B and D**). As a result the cell margin is thinner and the F-actin ring of the EVL and YSL appears more compact. Confocal images at higher magnification (40×) comparing (**C**) un-injected control and (**D**) Chp morphant stained with β-tubulin. Microtubule organization in Chp morphant was largely normal. Scale bars represent 40 µm in panels A-B and 20 µm in panels C-D.

Previous reports have documented the prominent organization of F-actin at the advancing edge of the YSL and EVL during epiboly [24], [32]. This band of F-actin is likely contributed primarily by the YSL, which leads the migration process, just beneath the EVL marginal region [24]. By confocal imaging, one can also find an F-actin margin at the DEL/EVL junction (Figure 3A). Although the leading edge actin band in Chp morphants was sometimes thinner (Figure 3B), there was no drastic loss of F-actin in this region. Based on the nuclei position in confocal analysis, the DEL cells were depleted from the leading edge region at 8 hpf (Figure 3B) relative to YSL and EVL nuclei. Thus movement of these deep cells is probably inhibited during vegetal pole migration. In addition there appeared to be a general delay of coordinated YSL/EVL migration relative to controls. As a result, the leading EVL cells were laterally expanded, perhaps reflecting a lack of coordinated movement of the more posterior cells. Two prominent microtubule arrays are found in epiboly; one with a dense network across the YSL and another array arranged in parallel along the animal-vegetal axis in yolk cytoplasmic layer [23]. The process of epiboly is affected when microtubules are disrupted; pregnenolone generated from cholesterol by Cyp11a1 is suggested to promote proper epiboly by maintaining polymerized microtubules [33]. We found that microtubule organization in Chp morphants was largely normal (Figures 3C and 3D).

### Chp is required for the normal organization of E-cadherin

**Figure 4:**
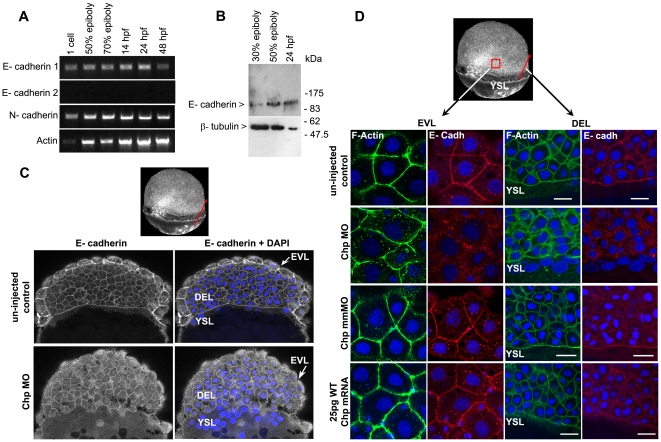
E-cadh is not maintained at the AJs in the absence of Chp. (**A**) RT-PCR products of zebrafish E-cadh1, E-cadh2 and N-cadherin (N-cadh) at the stages shown. E-cadh1 transcript was present throughout all stages however we did not detect expression of E-cadh2 (although it is represented 3 times in the EST database). N-cadh transcripts accumulate more significantly at 50% epiboly. Primers encoding the cytoplasmic domain of *E-cadh1* NM_131820, *E-cadh2* XM_690906 and *N-cadh* AF 418565 were used. (**B**) Immunoblot analysis of endogenous E-cadh at the stages indicated. The E-cadh antibody (BD Biosciences) detects a single E-cadh band (approx. 120kDa). (**C**) Low resolution image of phalloidin stained embryo of 60% epiboly, marked with red arrow representing the ‘y’ component in xy cross-section of the embryo (EVL→ DEL→ YSL) where the confocal image is taken. A middle sagittal plane of the embryo at 60% epiboly is derived from the Z stack. E-cadh was no longer maintained at the AJs in the EVL and was found predominately in cytoplasm. (**D**) Schematic diagram (3D view) of 60% epiboly. Red box indicates the lateral area of EVL and red arrow indicates the ‘y vector’ of the xy confocal slice. Confocal images (40× mag.) showing E-cadh co-localized with F-actin at the AJs between adjacent cells in the EVL and DEL. These observations were similar with the controls that were injected with 25 pg WT Chp mRNA alone and Chp mmMO. The images represent a stack of 10 images (each 0.5 µm); E-cadh staining was rarely detected at AJs in Chp morphant but intracellular signal was not diminished, suggesting E-cadh is mis-localized without Chp signaling. Identical loss of E-cadh was observed (data not shown) with a Mab that recognized the extracellular domain of E-cadh (ECM Biosciences; CM1681). Scale bars represent 20 µm.

In order to better assess the interplay between the cells in the three layers, we assessed Chp morphants at 60% epiboly for E-cadh; an important cell-cell junctional marker in early development [1]. In zebrafish, E-cadh is expressed maternally in all blastomeres. At the onset of gastrulation, E-cadh message accumulates in the region of the shield, which is the dorsal organizing center equivalent to Spemann's organizer (Babb et al., 2001). E-cadh plays a critical role in regulating cell-cell interactions during epiboly [4], [5]. Mutations of *E-cadh* termed *half baked* (*hab*) or *cdh1rk3* result in cells that failed to remain restricted in the exterior layer of epiblast after being radially intercalated from the interior layer [4], [5]. In the *cdh1rk3* mutant, defective adhesion between EVL and DEL cells was suggested to lead to delayed epiboly [4]. The dynamic remodeling of cell adhesiveness by E-cadh does indeed promote cell movement and tissue segregation [22]. At this stage, N-cadherin (N-cadh) is required for mesodermal germ layer development as evidenced by the *biber (bib)* mutant [34]. N-cadh is first expressed in the yolk cell at the time when the blastoderm uniformly expresses E-cadh [34]. In support of these findings, the mRNA levels of cadherins assessed by RT-PCR indicate both E-cadh and N-cadh are present early in development and thus epiboly (Figure 4A). We ruled out the involvement of a second zebrafish E-cadh transcript not previously described (cf. E-cadh-2 present in genomic and EST databases) since no transcript was detected at these stages.

Western blot analysis indicated the antibody recognizes the 120 kDa E-cadh present at 30% and 50% epiboly and at 24 hpf (Figure 4B). Prominent staining of E-cadh at the adherens junctions (AJs) at 60% epiboly was observed, particularly in the epithelial-like EVL as well as in the underlying DEL, and the margin of DEL/YSL layer (Figures 4C and 4D). This distribution of E-cadh at 60% epiboly was similar in un-injected controls, or embryos injected with a mismatch (mm)MO, or with 25 pg WT Chp mRNA (Figure 4D). Remarkably, Chp morphants consistently lacked normal cell surface E-cadh particularly in the EVL (Figures 4C, 4D and Figure S3) while the DEL E-cadh staining was more varied even though it was often absent from the DEL (Figures 4C, D). Junctional F-actin levels were largely unaffected (Figure 4D and Figure S3) although we noted that these junctions were always less organized consistent with the role of E-Cadh in F-actin organzation. The depressed E-cadh staining at EVL cell junctions in Chp morphants was confirmed with antibodies that recognized both extracellular and intracellular domains of E-cadh (see materials). Loss of E-cadh at AJs was accompanied by increased cytoplasmic puncta (Figure 4D): we conclude that junctional E-cadh requires Chp signaling to be maintained at the AJs. Perturbation of cell-cell junctions was accompanied by more irregular cell-cell junction in the EVL (Figure 4D). This indicates that Chp is needed to localize E-cadh at the AJs during the dynamic cell adhesion in epiboly. It is notable therefore that the Chp MO phenocopies *E-cadh* mutants [35], and suggests that the interplay between Chp effectors and E-cadh is key to understanding the regulation of cell behaviour at this early stage of development. Since we do not find any notable changes in E-cadh levels, it seems unlikely that Chp antagonizes an epithelial-mesenchymal transitions (EMT)- like pathway to down-regulate E-cadh in the embryo.

### Chp signaling maintains both E-cadh and β-Catenin at cell-cell junctions

**Figure 5:**
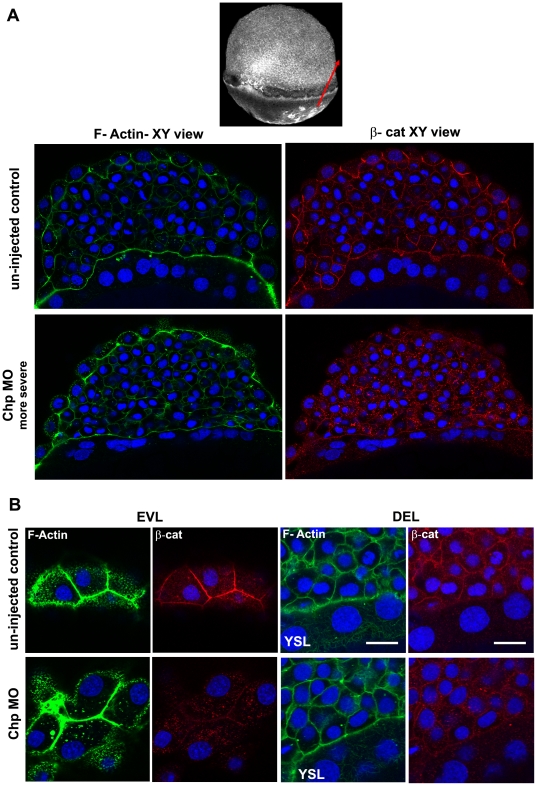
Localization of β-cat at the AJs requires Chp signaling. (**A**) A middle sagittal plane of the embryo at 60% epiboly immunostained with β-cat is also derived from the Z stack. β-cat was no longer maintained at the AJs in the EVL and was found predominately in cytoplasm. Scale bars represent 20 µm. (**B**) Immunostaining of β-cat comparing the EVL and DEL of un– injected control and Chp morphant. Levels of anti-β-cat staining at AJs were severely reduced in Chp morphants. The delocalized β-cat protein was detected within intracellular structures, but no nuclear enrichment was observed relative to controls.

The cytoplasmic tails of cadherins are suggested to be anchored to the underlying actin cytoskeleton via catenins [12] although some questions have been raised to this model [36], [37]. In order to assess the contribution of E-cadh to catenin localization, we immuno-stained embryo for β-catenin (β-cat), which binds to a conserved region of conventional cadherins [12], in control and Chp morphant embryos. The level of β-cat staining at cell junctions was severely depleted with loss of Chp in the morphants (Figure 5A and 5B); the β-cat was instead found within intracellular structures, but no nuclear enrichment of β-cat was observed in either case. Taken together we conclude that Chp signaling is required to maintain E-cadh and the β-cat complex and indicating that N-cadh does not compensate at this stage in spite of mRNA being detectable by RT-PCR. These findings represent the first indication that specific signaling by a Rho protein is required to maintain E-cadh/ β-cat at the AJs. It seems unlikely that this pathway controls activity of constitutive membrane recycling machinery such as Rab5 [38] but could act by coupling between E-cadh and this machinery. We note that the interplay of Rho proteins and E-cadh has been found for related cellular processes, for example EMT [39]. In studies where the formation of E-cadh-mediated epithelial cells junctions stimulates the activities of Cdc42 [40] and Rac1 [41], an interaction between p120cat and Vav2, a GEF for multiple Rho proteins, may be involved [35].

This remodeling of the embryonic AJs involving the E-cadh is likely a consequence of altered rates of cadherin endocytosis or exocytosis. The AP-2 adaptor complex is a well conserved protein complex that participates in clathrin-mediated endocytosis [42]. Co-staining of either E-cadh or β-cat with AP-2, suggested a portion of the intracellular E-cadh and β-cat co-localized with the AP-2 vesicles in Chp morphant embryos (Figure S4). In the absence of Chp signaling, we also noted that more AP-2 cytoplasmic vesicles could be seen near the cell-cell junctions (Figure S4).

### Interfering with βPIX phenocopies Chp loss

**Figure 6:**
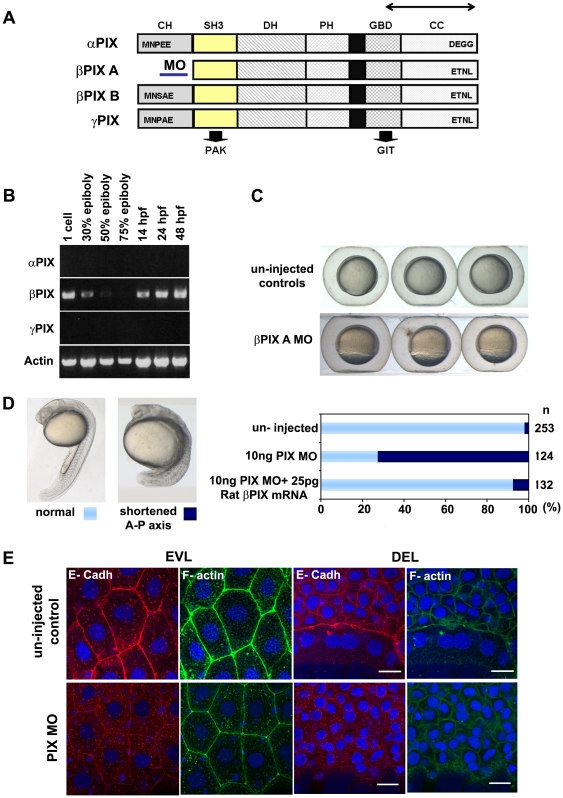
PIX is required to localize E-cadh at the AJs. (**A**) Schematic of zebrafish *PIX* isoforms designated *αPIX, βPIX-A, βPIX-B* [7] and the newly described *γPIX*. Arrows indicate the positions of the oligonucleotide primers used for RT-PCR. The position of the PIX-MO at the 5′ UTR of *βPIX-A* is indicated: this transcript encodes the smaller PIX isoform which is equivalent to the ubiquitous mammalian βPIX. SH3 domain in yellow and GBD binds to PAK and GIT respectively. (**B**) Transcript profile showing RT-PCR products for PIX at the developmental stages indicated. Primers cover essentially the same region of the PIX ORFs and therefore do not discriminate between the alternate spliced forms at the 5′ terminus. (**C**) PIX morphant embryos exhibit epibolic delay compared to un-injected controls at 8 hpf. (**D**) The typical phenotype of embryos depleted of βPIX-A at 24 hpf, exhibit shortened AP axes suggesting gastrulation defects. Phenotypic analysis showing significant rescue at 24 hpf after co-injection with 25 pg of rat βPIX mRNA. (**E**) Reduced cell junctional E-cadh signals in the EVL and DEL after PIX knock-down. The level of cortical F-actin (phalloidin) is similar to controls but the junctional network is more irregular. Intracellular E-cadh puncta suggest PIX functions downstream of Chp to maintain E-cadh at cell adhesions. Scale bars = 20 µm.

The downstream targets or effectors of Chp are not characterized, however our studies in mammalian cells (Manser unpublished data) suggested that a key Chp target is the serine/threonine kinase PAK1 that binds both to Rac1 and Cdc42 [43], and requires targeting via the multi-domain partner PIX [44], [45]. Although there are three zebrafish *PIX* genes (Figure 6A), we only detect mRNA expression of the orthologue of βPIX [7] at the stages considered here (Figure 6B). The PIX proteins from vertebrates and invertebrates contain an N-terminal SH3 domain that binds specifically to conventional PAKs [44]. The longer CH domain-containing alternate spliced form of zebrafish βPIX is essential for the integrity of the blood-brain barrier [7], as is the function of PAK2a [6]. The role of the ubiquitous βPIX-A (shorter isoform) has not been assessed; a third zebrafish gene product we designated as *γPIX* has not been described previously (Figure 6A). All PIX genes potentially encode longer proteins with an N-terminal CH domain or shorter versions which initiate just upstream of the SH3 domain (Figure 6A).

Rho GTPases such as Rac1 and Cdc42, and the critical PAK partners; PIX and GIT proteins positively contribute to PAK1 activation [43], [46]. Although the PIX GEF domain can act on Rac1 *in vivo* [44], [45], the Rho GEF domain is essentially inactive when tested *in vitro* (Manser, unpublished). One possibility is that the recruitment of smgGDS to the coiled-coil region of PIX [47] instead activates Rac1. The PAK1 family kinases contain auto-inhibitory region which packs against the kinase domain to maintain a normally inactive state. Previous work has established that βPIX forms a tight complex with the ArfGAP GIT1 to allow recruitment of the kinase to adhesion complexes and the centrosome [48]. It seemed likely that zebrafish Chp could be involved in activating PAK kinase(s) at this early stage of development and that this would require its recruitment via PIX. Knockdown of the smaller βPIX isoform (βPIXA) using the MO targeted at the 5′ UTR, yielded embryos with delayed epiboly (Figure 6C) and with shortened A-P axes at 24 hpf (Figure 6D). This phenotype could be rescued by co-injection of mRNA encoding rat βPIX (Figure 6D). The epibolic delay associated with treatment with PIX MO might have a range of underlying causes. However the de-localization of E-cadh from the AJs in the EVL and DEL, and the similarity with E-cadh loss of function suggests this is critical [4], [5]. The βPIX morphants showed that at 60% epiboly, E-cadh is predominantly cytoplasmic in both the outer EVL cells and the DEL, again with the junctional actin cytoskeleton remaining relatively unaffected (Figure 6E).

### Chp is an upstream activator of PAK1 at cell adhesions

**Figure 7:**
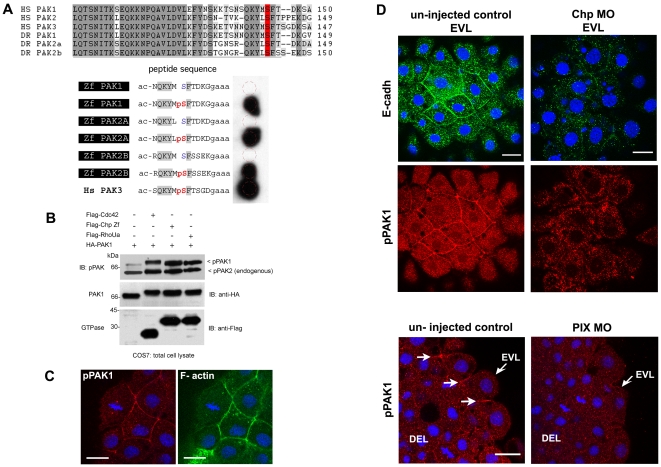
PAK lies downstream of Chp signaling and active kinase co-localizes with E-cadh. (**A**) Characterization of the phospho-PAK (pPAK) antibody. Alignment of auto-inhibitory domain (AID) of human (Hs) and zebrafish (Zf) PAKs: HsPak1, NP_001122092.1; HsPak2, NP_002568.2; HsPak3, NP_002569.1; ZfPak1, NP_958485.1; ZfPak2a, NP_001002717; and ZfPak2b, NP_001020627.1. Dark and light grey shaded sequences represent identical and conserved amino acids; the serine residue highlighted in red is the phosphorylated site recognized by the rabbit anti-pPAK144 antibody. Further characterization of this new antibody will be presented elsewhere; it is ∼10 times more sensitive than the anti-PAK1 pSer199 described previously in zebrafish [85]. Anti-pS144PAK1 recognizes all zebrafish PAK isoforms as assessed by synthetic phospho-peptides representing zebrafish PAK sequences. The peptides were synthesized *in situ* on cellulose (Jerinini) with a 3 amino-acid linker at the C-terminal end, and N-terminally amidated. The filter was blocked with BSA, and probed with anti-pPAK and HRP-anti-rabbit IgG antibodies both at 1∶2000 (30 min each, with 3×10 min washes). Human PAK3 (pS139) is shown as a positive control. (**B**) Constitutively active Chp (G38V) can activate PAK1. Active Cdc42(G12V), Chp(G38V) and RhoUa(Q104L) were cloned in the mammalian expression vector pXJ-Flag (with CMV promoter) and co-expressed with HA-tagged PAK1 in COS-7 cells. The activation of PAK1 is indicated by an upshift in the PAK1 band and by PAK1 phosphorylation on Ser144. (**C**) Activated pPAK1 was detected at the centrosome of mitotic cell (as previously reported in mammalian cell culture) and can be found at the cell junctions of envelope cells (EVL), but not on the junctions of deeper cells. (**D**) E-cadh colocalizes with pPAK1 at the AJs of the EVL but not in the cytoplasmic puncta. Both were reduced at the AJs of the EVL in Chp morphants. Junctional pPAK signal is reduced in the envelope cells in PIX morphants. Typical stainings of control and PIX morphant embryos. Both images represent a stack of 3 confocal images, collected under the same laser and gain settings, and at equivalent positions in the embryo. White arrows represent junctional pPAK1. Scale bars represent 20 µm.

The conventional PAK family in zebrafish is represented by at least 3 isoforms where Zf Pak1 is the most closely related to mammalian PAK1 (cf. Figure 7A); knock down of PAK2a and PAK2b and their respective expression pattern in fish indicates these proteins are key in the vasculature and promote integrity of the blood-brain-barrier [6], [7]. In order to detect active PAK, we used an antibody specific towards a conserved auto-phosphorylation site equivalent to PAK1-pS144 (Figure 7A) and located towards the C-terminal end of the auto-inhibitory domain (AID), adjacent to Cdc42/Rac interactive binding (CRIB) domain. Using synthetic peptides corresponding to sequences found in the three fish PAK isoforms, the antibody is shown to recognize all these phosphorylated forms (Figure 7A). Human Chp is known to activate PAK1 [27], and the ability of zebrafish Chp to activate PAK1 was therefore tested; Chp(G38V) when co-expressed with PAK1 in COS7 cells undergoes a mobility shift consistent with kinase activation, and is accompanied by increased levels of phospho-S144 (Figure 7B), as for human Cdc42(G12V) as well as the Chp related RhoUa. As the CRIB region of human and zebrafish PAKs are essentially identical, we can conclude that Chp is indeed able to act upstream of PAKs in zebrafish.

Given the likely involvement of Chp in PAK activation, we tested whether active PAK is detected in these early embryos using the anti-pS144 antibody. It should be noted that when phosphorylated, the site contributes to PAK1 activation as well as the activation loop threonine [49]. Active PAK is found at the cell-cell junctions in the EVL (Figure 7C), while the active kinase is not detected here in either the Chp or PIX morphants (Figures 7D), although the cortical actin cytoskeleton is relatively normal. The phospho-PAK was observed to be enriched on centrosomes (Figure 7C) as previously described in cell culture [48]. We note that active PAK did not co-localize with the intracellular E-cadh (Figure 7D). These observations strongly suggest that active PAK1 acts locally at AJs to promote the localization of E-cadh. This represents the earliest role described for the PAK-PIX complex, and indicates that Chp plays an important early role in the development of vertebrates. The reported role of Rac1-PAK1 in promoting disassembly of E-cadh junctions in adult differentiated mammalian keratinocytes [50] is opposite to what we observe.

**Figure 8:**
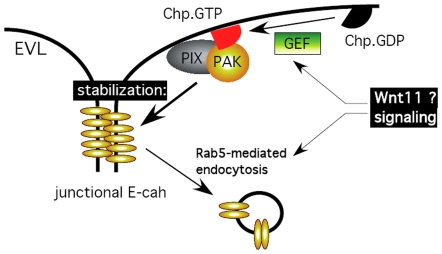
Model of the Chp-PAK-PIX mediated pathway uncovered in this study, and its possible location with reference to other components controlling E-cadh localization at the EVL of epiboly. Wnt11 has been shown to control E-cadh via a Rab5 sensitive pathway [3].

With respect to upstream signaling, Wnt11 plays a central role in tissue morphogenesis during vertebrate gastrulation [3], [51], [52]. It acts in part by regulating the cohesion of mesodermal and endodermal (mesendodermal) progenitor cells [3]. The involvement of the generic endocytic regulator Rab5 is suggested since blocking Rab5c activity in wild-type embryos phenocopies slb/wnt11 mutants, although one would anticipate that many cell surface receptors systems are thereby perturbed. That being said, enhanced Rab5c activity in slb/wnt11 mutant embryos can rescue the mutant phenotype, probably via stabilization of E-cadh localization. This would suggest that the Chp pathway acts somewhere downstream of this Wnt11 pathway. A model depicting these interactions is presented in Figure 8 and provides us with a platform for further investigation.

## Discussion

### Pathways involved in regulating E-cadherin localization

E-cadh is one of the most important cell-cell adhesion receptors involved in tissue morphogenesis and maintenance of epithelial tissue integrity [12], [53]. Loss of E-cadh function has emerged as a key event for epithelial invasion and metastasis [54]. E-cadh expression becomes down-regulated when epithelial cells acquire motile and invasive characteristics particularly in embryonic development [55]. Rac1 activity can perturb the distribution of E-cadh at junctions in different cell types [56], [57], [58]. Other surface receptors, such as integrins, are not removed from cell-cell contacts with the same time frame as cadherins [59]. The Rac1- dependent disruption of junctions has been shown during intestinal epithelia differentiation [60], salivary gland morphogenesis [61] and tracheal tubulogenesis [62].

During clathrin-mediated endocytosis, membrane constituents such as E-cadh are transported via endocytic vesicles into early endosomes. Wnt11 signaling could be required for the endocytosis of E-cadh that involves Rab5c [3]. Clathrin- and Rab5-mediated endocytosis also participate in Rac1-GTP induction by various stimuli where Rac1 activation is proposed to take place on early endosomes; this Rab5-to-Rac pathway is invoked for primordial germinal cells migration zebrafish development [63]. Nonetheless, it is unclear how regulating Rab5 *per se* could generate a specific signal to relocate either Rac1 or E-cadh. The observations in this study which show that Chp signaling is required for proper E-cadh localization via PAK and PIX, strongly suggests *in vivo* activation of the kinase downstream of Chp. With respect to Rac1-PAK signaling, loss of the RacGAP chimerin is characterized by the development of round somites, lack of yolk extension, and a kinked posterior notochord [64]. These zebrafish chimerin morphants also show Rac hyper-activation and more rapid epiboly, thus suggesting Rac1 is a key player in leading edge movement.

In epithelial cells, activated Arf6 has been shown to promote the disassembly of AJs [65], [66]. Conversely, hydrolysis of GTP on Arf6 promotes the re-distribution of E-cadh to assemble AJs [67]. GIT1 is a PIP3-stimulated GAP for ARF6 [68], therefore GIT1 is a potential candidate effector in the βPIX/PAK1 pathway since it mediates localization of these proteins to cellular sites such as adhesion complexes and the centrosome [48]. In Drosophila, the absence of either Cdc42, Par6 or aPKC in the Par complex resulted in the intra- cellular localization of E- cadh, thereby disrupt the AJs in epithelial cells [69]. In another similar case, the affected cells could even undergo apical constrictions and eventually delaminated [70]. Both studies have revealed the regulators including CIP4, WASp, Arp2/3 and Dynamin were involved in the endocytic machinery mediated by E-cadh to maintain the stability and plasticity of *Drosophila* AJs.

### Rho GTPases and cell-cell junctions

Regulation of E-cadh-mediated cell-cell adhesion involves the activities of Cdc42, Rac and RhoA to organize local actin [40], [41], [56], [71], [72], [73], [74]. One study suggests this mechanism is essential in the movement of zebrafish germ cells *in vivo* [75]. However in this study, we have found that Chp is required to localize the junctional E-cadh and as a result β-cat at the AJs in the cells that make up the EVL and DEL (Figure 5). Nonetheless the fact that Chp expression does not appear until 30% epiboly suggests that at earlier times, alternate pathways might operate to localize E-cadh. In early E-cadh MO-injected embryos, the cleavage plane orientation between blastomeres is irregular, and adhesion defects prevent normal cell compaction [1] in contrast to the Chp morphants which are normal with respect to the early cleavage events, consistent with the lack of Chp mRNA at this stage (Figure 1B). At ∼50–90% epiboly, Chp protein (Figure 2C) and mRNA (Figure 1C) is expressed in all cell layers and at this stage required for E-cadh- mediated adhesion leading to vegetal movement of the EVL, DEL and YSL to complete epiboly. One would anticipate however that the GTP-bound (active) Chp would be spatially organized. We find that Chp MO injection into the YSL at 30% epiboly is also capable of causing epobolic defects (data not shown). It is suggested that in *Fundulus* embryos the YSL drives epiboly by towing the EVL during the vegetal pole migration, even in the absence of the blastoderm [21]. In our studies, Chp knock down leads to a delayed migration of the YSL and EVL but an absence of DEL cells is noted at the migrating front (Figure 3), although the leading edge actin is essentially normal. In this context, we have not tested if Rac1 is required for the formation of this F-actin structure even though it is suggested from other studies in *Drosophila* dorsal closure [76]. The Chp phenotype is entirely consistent with loss of E-cadh leading to both delayed epiboly and gastrulation [1], [4], [5]. During epiboly, E-cadh RNA forms a gradient, with low levels in the deep cell layers, and increasing levels towards the EVL [5]. A minor effect on F-actin structures is observed in MZ*cdh1rk3* mutant embryos where the location of the DEL margin is displaced relative to the EVL margin. Mediolateral intercalation might be affected in these mutant embryos; this drives convergence and extension (CE) in the embryos and therefore these E-cadh-mediated cell adhesion defects occur simultaneously with epiboly. The defective movement of deep cells has been monitored in *E-cadh* mutants [4], [5]. The adhesion between the deep cells (DCs) and the EVL was most disrupted while that between the DCs is less unaffected in the MZ*cdh1rk3* mutant or E-cadh morphant embryos. The organization of microtubules remains unaffected in Chp morphants (Figure 3D) consistent with a minor role for cadherins in regulating microtubules [77].

### What are the signals downstream of Chp

It is clear that PAKs operate downstream of multiple Cdc42- and Rac1-like proteins. *E-cadh* knockout in mice produces early embryonic lethality due to a requirement for the protein in proper development of the extra-embryonic epithelial trophectoderm [78]. That E-cadh-mediated cell-cell adhesion lies downstream of a vertebrate-specific Rho GTPase is somewhat unexpected given the conserved processes of gastrulation. In mammalian systems, αPIX has been shown to be important for function of the immune system [79], but *βPIX* knockout has not been reported. Although PIX is present in Drosophila, mutants in *dPix* primarily affect later developmental stages, including postsynaptic development of the neuromuscular junction [67] although the maternal contribution has not been assessed. It is also noted that the major partner for PIX, an Arf-GAP GIT1 is required for proper muscle attachment which links the protein to integrin signaling [80]. None of these functions appear to be informative with respect to the role of the vertebrate orthologues in early development.

With respect to other roles for vertebrate PAK signaling, a hypomorphic mutation of Pak2a causes cerebral hemorrhage in zebrafish at 48 hpf [6]. This phenotype is consistent with an interdependence of PAK with PIX, since loss of a longer transcript of βPIX [7] gives a virtually identical phenotype. In the light of our findings, PIX and PAK could function to properly localize cadherins in the brain vasculature: cadherin-10 is an important blood-brain barrier adhesion protein [81]. It is not clear which zebrafish PAK isoforms are expressed during early development; although PAK2-null mice are embryonic lethal [82], PAK1 or PAK3 loss in mice has rather minor developmental defects [83], [84].

How might cadh localization be regulated by the activities of Chp? Although we reported that PIX is a weak activator of Cdc42 and Rac when over-expressed in cells [44], the protein has essentially no *in vitro* activity and does not act on Chp, itself thus ruling out a positive feedback loop (unpublished data). It has recently been suggested that E-cadh is responsible for the organization of fibronectin in *Xenopus* embyos [85]. In this proposed pathway, myosin II light chain (MLC2) phosphorylation lies downstream of Rac1 (perhaps via PAK); inhibition of Rac1 or PAK blocked cortical actin assembly of fibronectin fibrils in the extracellular matrix.

In conclusion, this work provides evidence for Chp function in regulating E-cadh early in vertebrate development, and suggests a similar role at later developmental stages. Non-canonical Wnt signaling in zebrafish initiates the cellular rearrangements and migration that contribute to convergent extension of involuting mesoderm that involves multiple Rho GTPases. This is initiated by the Wnt11 receptor Frizzled 7 that activates a PKC-dependent signaling pathway to promote Cdc42 activation [86]. As for the planar cell polarity (PCP) signaling pathway in *Drosophila*, vertebrate Wnt signals involve the conserved GTPases Cdc42 and RhoA via the polarity complex and the formin DAAM1 respectively [87], [88]. Our data suggest that morphology and migratory behaviors in vertebrates involve an additional Rho GTPase Chp during gastrulation. It will be interesting to see if alternate Cdc42-like GTPases signal via PAK/PIX pathway to regulate E-cadh in other epithelial containing tissues during development.

## Materials and Methods

### Mammalian cell transfection

COS-7 cells (ATCC) were grown in Dullbecco's modified Eagle's medium supplemented with 10% fetal bovine serum (FBS). Anti-Flag M2 antibody and M2-agarose, D-Biotin and Streptavidin sepharose were from Sigma-Aldrich. Cells transfection was performed with Lipofectamine 2000 according to the manufacturers protocol (Invitrogen). After 16 h cells were lysed in buffer containing 50 mM Tris-HCl, pH 7.4, 150 mM NaCl, 2 mM MgCl_2_, 0.5% Trition X-100, 10% glycerol, and protease inhibitor cocktail (Roche), and cleared by centrifugation at 12K, 10 min. The cell lysates (40 ug per lane) were separated by SDS-PAGE using 10% acrylamide and transferred to PVDF (Millipore).

### Generation of anti-polyclonal antibody

Serum was collected 16 weeks after three injections of rabbits with the KLH-peptide complex containing the amino terminus (MPPQMDYFYHESRVP) of zebrafish Chp (Genemed Synthesis Inc.). Sera from two animals termed Pab1 and Pab2 were purified on Sepharose-coupled peptide and eluted in100 mM glycine-HCl (pH 2.5), 0.05% Triton X-100 and immediately neutralized with Tris/HCl (pH 8.5) and positive fractions tested by Western blot analysis.

### RT-PCR, gene cloning and site directed mutagenesis

Total RNAs were extracted and isolated from various stages of zebrafish embryos using Qiagen RNeasey mini kit and QIAshredder kit. The RT-PCR reactions were performed using M-MuLV reverse transcriptase (NEB). The cDNAs for the zebrafish genes were cloned into pXJ vector using primers flanking the coding sequence. Reference sequences in the GenBank database: Cdc42a; AY865566, Cdc42b; XM_678979, Cdc42c; AY865567, RhoV; NM_001012250 and PIX; DQ656108 correspond with the genes that were cloned. Dominant inhibitory Zf-Chp(T43N) and constitutively active versions of Zf-Chp(G38V), RhoUa(Q104L), and Cdc42(G12V) were generated using QuikChange site-directed mutagenesis kit (Stratagene). Sense and anti-sense primers used are given in the Material S1. Constructs were confirmed by DNA sequencing.

### In-vitro synthesis of RNA

pXJ-FLAG Chp or pXJ-HA βPIX plasmids (Manser et al., 1998) were linearized with *Sal*I and ethanol purified after phenol/chloroform extraction. The capped synthetic sense mRNAs were generated by using 0.5–1 µg linearized DNA, 1 mM ribonucleoside triphosphate set (Roche) with m7G(5′)ppp(5′)G (0.5 mM) and T7 RNA polymerase (20 units) in a total reaction volume of 50 µl. Synthesis was carried out at 37°C for 1 h, and subsequently treated with DNase for 30 min. The RNA was precipitated using lithium chloride (4 M) and the concentration determined by absorbance at 260 nm.

### Whole mount RNA in-situ hybridization probes encoding

Chd, Gsc and Ntl in PCS vector (David Turner and Ralph Rupp,) were linearized and DIG-labelled RNA probes (Roche) were synthesized using DIG-dUTP. The DIG labeling reaction was incubated at 37°C for 2 hours and subsequently treated with DNase at 37°C for 30 minutes. RNA probes were precipitated using lithium chloride and resuspended in water. *In-situ* hybridization on the whole embryo was performed as described by Shamim et al. (1999) and NBT: BCIP (Roche) was used to detect the reaction.

### Morpholino injection and rescue experiment

Microinjection of zebrafish embryos was performed at one cell stage using IM300 microinjector (Narishige). The embryos were incubated at 28°C and transferred to 24°C after gastrulation. The phenotypes of embryos were examined and scored after 24 hours. The staging series of zebrafish embryonic development was based on the studies by [89]. Two morpholino oligonucleotides (Gene Tools Inc.) were as follows: *Chp/RhoV* MO1 (5′-GAGGTGGCATGTC-CGACAACTAAAG-3′) and *Chp/RhoV* MO2 (5′- CAGGGAGAAGCGT-CCTCAGGAAAAC-3′) were designed to target against the *Chp/RhoV* gene. *Chp/RhoV* mmMO (5′-GAcGTGcCATcTCgG-ACAAgTAAAG-3′) contained five mismatches served as control. 5 ng of *Chp/RhoV* MO1 or MO2 was sufficient to yield embryos with delayed epiboly whereas *Chp/RhoV* mmMO gave no defect. PIX MO (5′-GTACTGGTTGAA-CTTGTGCCTGGAG-3′) is targeted against the 5′UTR at the βPIX A and 10 ng gave shortened A-P axes embryos at 24 hpf. The synthetic capped RNA was titrated to determine concentrations with minimal embryo defects. Subsequently, co-injection of MO and synthetic capped RNA was tested in rescue experiments.

### Immuno-histochemistry and microscopy

Rabbit anti-peptide antibodies pooled from the affinity purified fractions directed towards zebrafish Chp were used at 1∶500. Mouse monoclonal anti-β-tubulin (Chemicon), anti-E-cadherin (BD Biosciences 610182), anti-β-catenin (BD Biosciences, 610154) and goat polyclonal anti- α-adaptin2 (M16) (Santa Cruz, sc6422) were used at 1∶500. Embryos were fixed in 4% formaldehyde in PBS overnight at 4°C, and dechorionated in 1X PBS. Incubations were at room temperature unless indicated otherwise. Embryos were permeablized and blocked in phosphate buffered saline-0.1% Triton X-100 (PBS-Tx) containing 1% goat serum for 4 h. Primary antibodies were added at 4°C overnight and washed in 1X PBS-Tx (3×30 min). Alexafluor 594 anti-rabbit and anti-mouse (Molecular Probes) were used at 1∶1000 in 1% goat serum. Alexafluor 488 Phalloidin (Molecular Probes) and DAPI (Sigma) were each diluted at 1∶500 from stock and incubated together with the secondary antibody for 2 h. The stained embryos were then washed in 1X PBS-Tx (3×30 min). They were mounted in 80% glyercol on the depression slides and imagining was performed using Olympus Fluoview 1000 confocal microscope: serial Z-stack sections were taken with 0.5 µm step size.

## Supporting Information

Figure S1 Amino acid alignment of zebrafish (Dr) and human (Hs) Cdc42 family members. Sequences of Cdc42a, Cdc42b, Cdc42c and Chp (Dr) were aligned with human Cdc42 and Chp using ClustalW. Accession numbers of corresponding to human Cdc42 isoforms 1, 2 and Chp are NP_001782.1, NP_426359.1 and NP_598378 respectively. The N-terminal extension at the amino terminus end of Chp-Hs is shorter in the zebrafish form and not conserved. The Chp proteins lack a canonical CAAX motif but rather CFI or CFV. Conserved residues among all Cdc42 proteins are highlighted in grey, and identical residues comparing human and fish Chp are marked in yellow. Positions containing identical residues across all proteins are indicated by a star.
(0.62 MB TIF)

Figure S2 Early patterning and organizer specification of un-injected control and Chp morphants. WISH for mesodermal markers; (A-E) chordin (Chd), (F-J) goosecoid (Gsc) and (K-O) no tail (Ntl) in un-injected controls, Chp morphants or embryos injected with Chp(T43N). Reduced activity of Chp did not affect the expressions of Ntl, Gsc and Chd at 50% and 70% epiboly although the overall morphology of the mutants is affected. Panels A-I are embryos at the shield stage. Panels J-O are embryos at 70% epiboly. Panels A-J provides dorsal midline views, and panels K-O lateral views.
(7.72 MB TIF)

Figure S3 Chp MO1 rescue by synthetic mRNA co-injection. Chp MO1 conjugated with fluorescein blocks E-cadh localization to AJs in EVL at 60% epiboly. The panels show typical phenotype for rescued of E-Cadh localization by the co-injection with 25pg Chp mRNA. Scale bars represent 20 µm.
(4.94 MB TIF)

Figure S4 A portion of E-cadh co- localizes with intracellular AP-2 vesicles. Confocal images (zoomed) of E-cadh with AP-2 in EVL cells. The un-injected controls and Chp MO2 injected embryos are compared. The image is a single confocal slice of 0.5 µm step size. The loss of the Chp signal leads to E-cadh depletion from AJs and becoming associated primarily with intracellular AP-2 vesicles clustered near the AJs. Scale bars represent 20 µm.
(6.90 MB TIF)

Material S1 Primer sequences.
(0.04 MB DOC)
