# Supplementary figures and images for: A Vertebrate-Specific Chp-PAK-PIX Pathway Maintains E-Cadherin at Adherens Junctions during Zebrafish Epiboly
Source: PLoS One. 2010 Apr 12;5(4):e10125. doi: 10.1371/journal.pone.0010125 (PMC2853574; doi:10.1371/journal.pone.0010125)

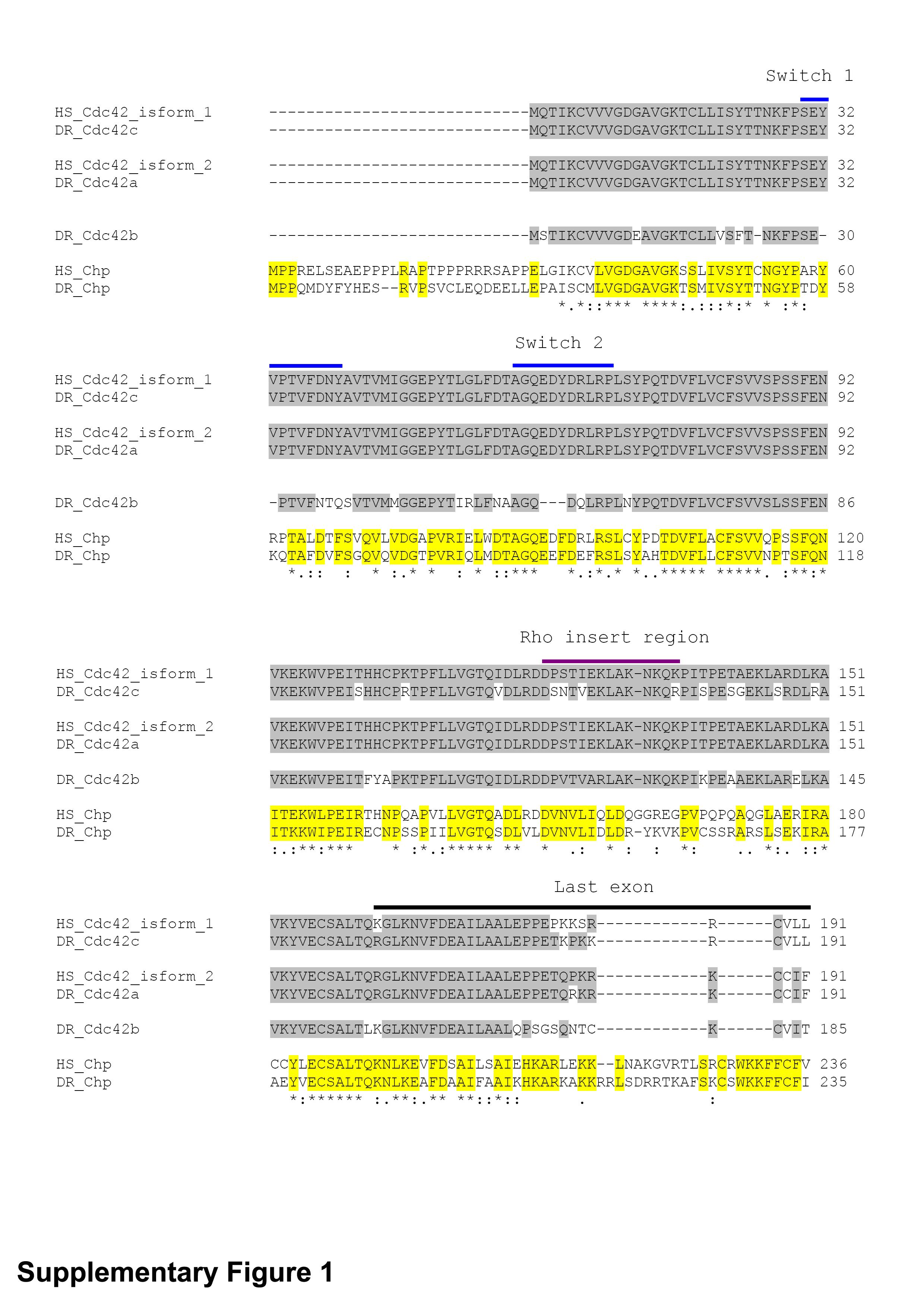

Supplement: Figure S1 — Amino acid alignment of zebrafish (Dr) and human (Hs) Cdc42 family members. Sequences of Cdc42a, Cdc42b, Cdc42c and Chp (Dr) were aligned with human Cdc42 and Chp using ClustalW. Accession numbers of corresponding to human Cdc42 isoforms 1, 2 and Chp are NP_001782.1, NP_426359.1 and NP_598378 respectively. The N-terminal extension at the amino terminus end of Chp-Hs is shorter in the zebrafish form and not conserved. The Chp proteins lack a canonical CAAX motif but rather CFI or CFV. Conserved residues among all Cdc42 proteins are highlighted in grey, and identical residues comparing human and fish Chp are marked in yellow. Positions containing identical residues across all proteins are indicated by a star. (0.62 MB TIF) [file pone.0010125.s001.tif]

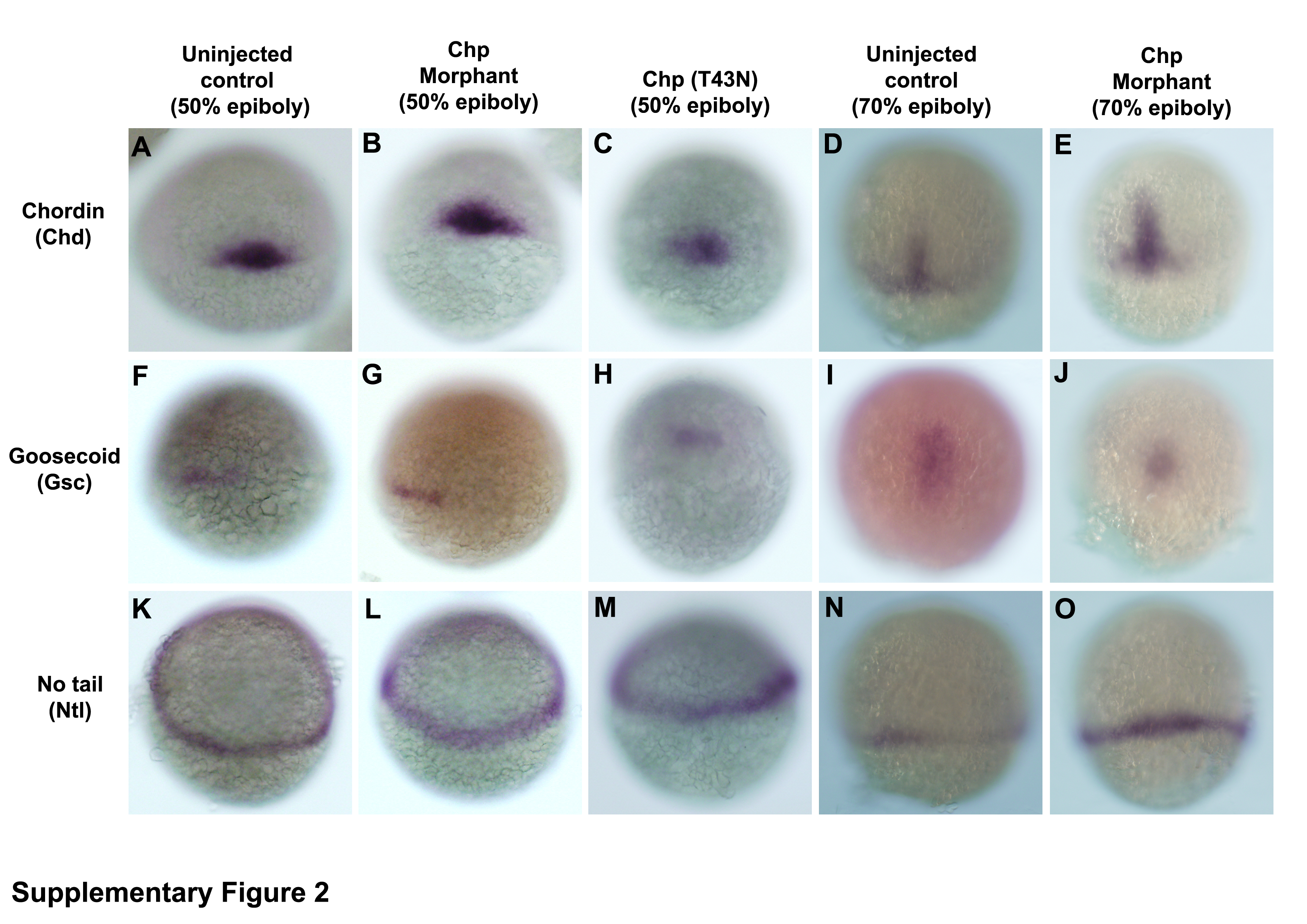

Supplement: Figure S2 — Early patterning and organizer specification of un-injected control and Chp morphants. WISH for mesodermal markers; (A-E) chordin (Chd), (F-J) goosecoid (Gsc) and (K-O) no tail (Ntl) in un-injected controls, Chp morphants or embryos injected with Chp(T43N). Reduced activity of Chp did not affect the expressions of Ntl, Gsc and Chd at 50% and 70% epiboly although the overall morphology of the mutants is affected. Panels A-I are embryos at the shield stage. Panels J-O are embryos at 70% epiboly. Panels A-J provides dorsal midline views, and panels K-O lateral views. (7.72 MB TIF) [file pone.0010125.s002.tif]

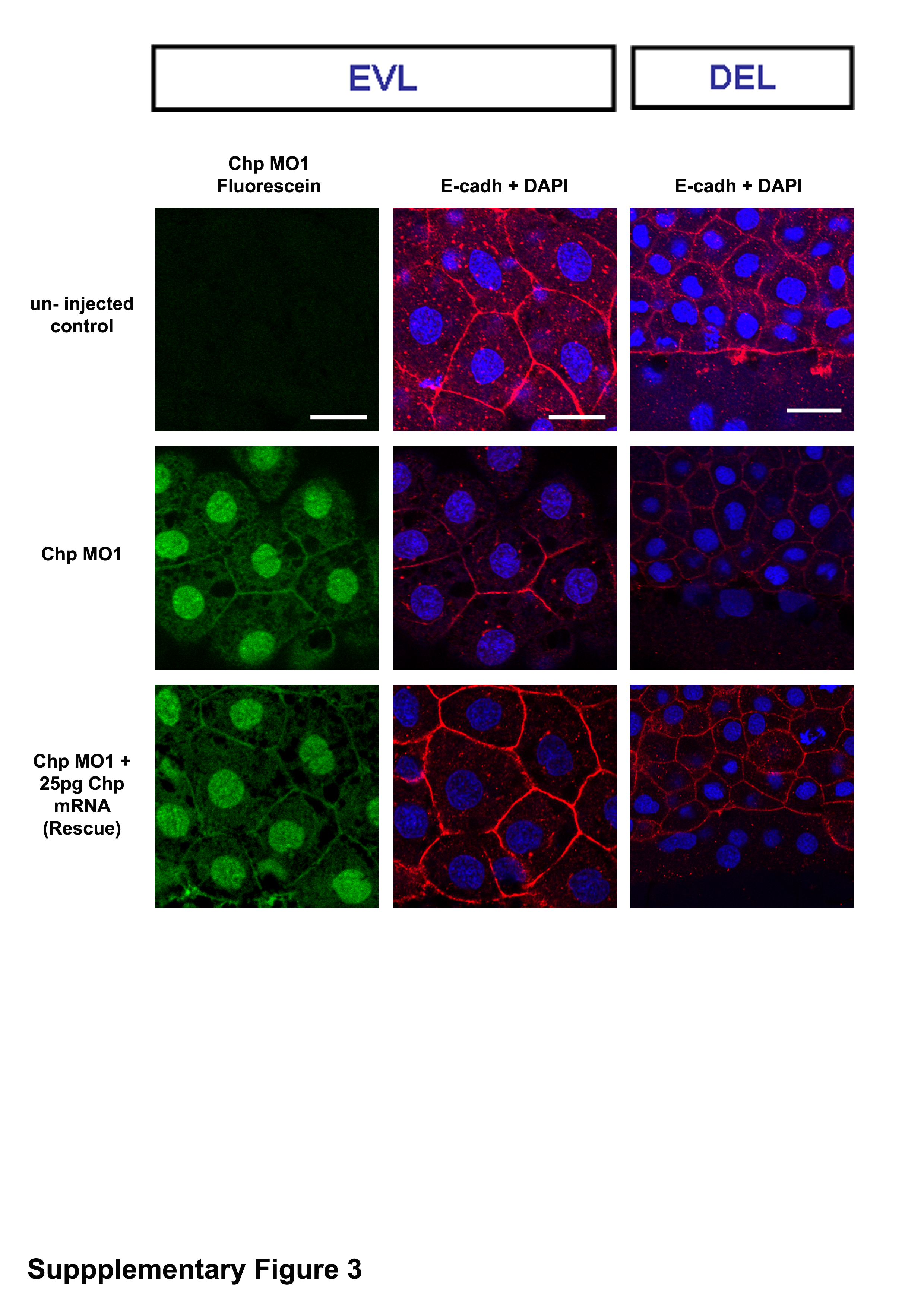

Supplement: Figure S3 — Chp MO1 rescue by synthetic mRNA co-injection. Chp MO1 conjugated with fluorescein blocks E-cadh localization to AJs in EVL at 60% epiboly. The panels show typical phenotype for rescued of E-Cadh localization by the co-injection with 25pg Chp mRNA. Scale bars represent 20 µm. (4.94 MB TIF) [file pone.0010125.s003.tif]

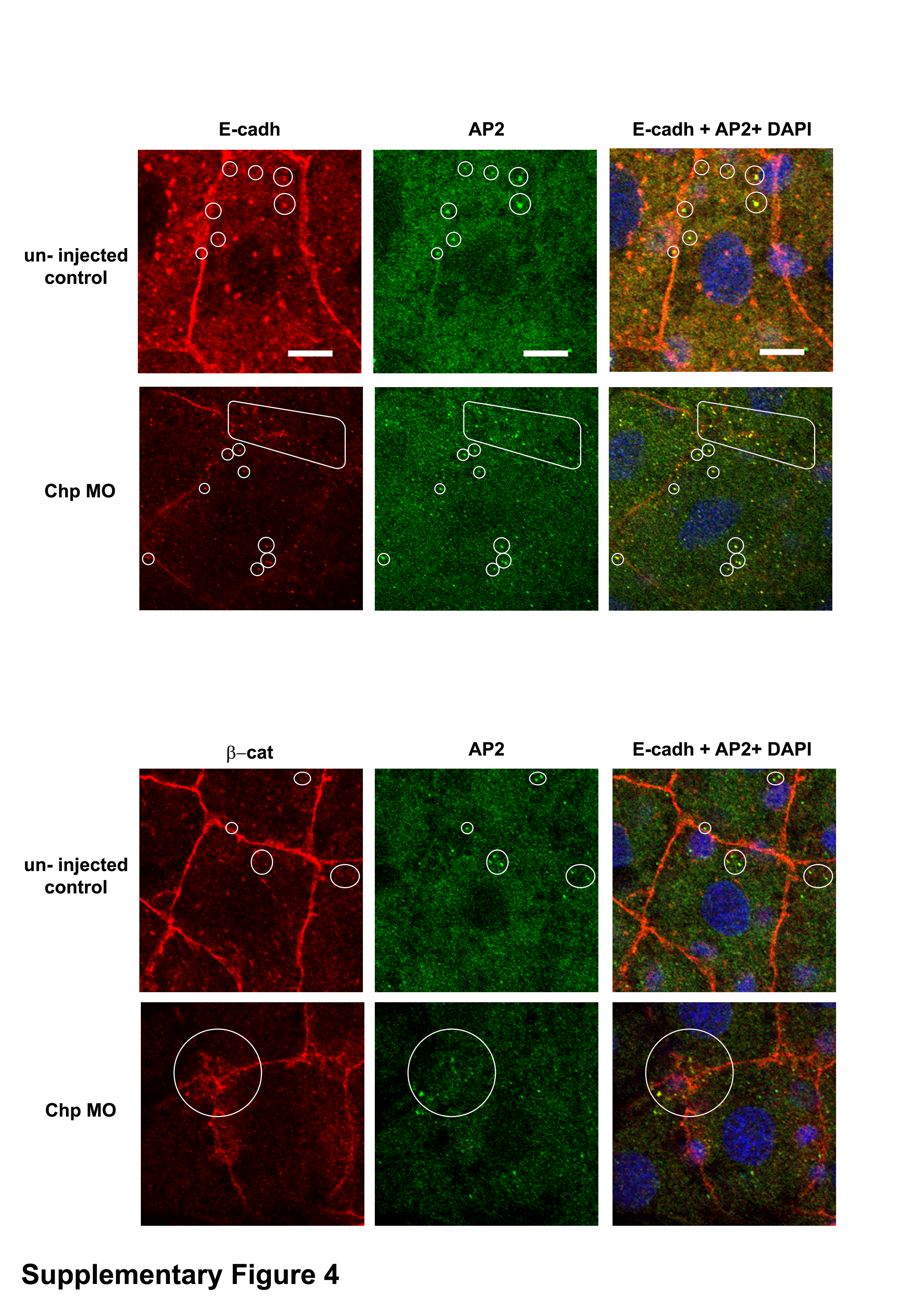

Supplement: Figure S4 — A portion of E-cadh co- localizes with intracellular AP-2 vesicles. Confocal images (zoomed) of E-cadh with AP-2 in EVL cells. The un-injected controls and Chp MO2 injected embryos are compared. The image is a single confocal slice of 0.5 µm step size. The loss of the Chp signal leads to E-cadh depletion from AJs and becoming associated primarily with intracellular AP-2 vesicles clustered near the AJs. Scale bars represent 20 µm. (6.90 MB TIF) [file pone.0010125.s004.tif]
