# Supplementary material for: A Vertebrate-Specific Chp-PAK-PIX Pathway Maintains E-Cadherin at Adherens Junctions during Zebrafish Epiboly
Source: PLoS One. 2010 Apr 12;5(4):e10125. doi: 10.1371/journal.pone.0010125 (PMC2853574; doi:10.1371/journal.pone.0010125)
Supplement: Material S1 — Primer sequences. (0.04 MB DOC) [file pone.0010125.s005.doc]

**MATERIAL S1**

1. Primer sequence

| **Primer** | **Sequence 5’ –> 3’** |
| --- | --- |
| **Cdc42a Forward** | **ATGCAGACGATCAAGTGCGTCGTTG** |
| **Cdc42a Reverse** | **GAAAGTGTGGGGAGTTAGAAAGAGA** |
| **Cdc42b Forward** | **ATGTCCACAATTAAATGTGTGGTAG** |
| **Cdc42b Reverse** | **TCATGTGATGACACATTTGCATGTG** |
| **Cdc42c Forward** | **ATGCAGACCATAAAGTGTGTGGTGG** |
| **Cdc42c Reverse** | **CTACAAAAGGACGCAGCGCTTCTTG** |
| **Rhov/ Chp Forward** | **ATGCCACCTCAAATGGATTACTTTT** |
| **Rhov/ Chp Reverse** | **TCAGATGAAGCAGAAGAATTTTTTC** |
| **β-actin Forward** | **CAACGGCTCCGGCATGTG** |
| **β-actin Reverse** | **TGCCAGGGTACATGGTGG** |
| **αPIX 1655 Forward** | **ATGTTAAACCTTTAACCATGCCTGG** |
| **αPIX Reverse** | **TTAGTGACCTCCTTCATCCCAGGAA** |
| **γPIX 1695 Forward** | **ACAGAGTGTGCCCTGCCATACACTG** |
| **γPIX Reverse** | **TCACAAATTGGTTTCATCCCAGGACGGGTC** |
| **βPIX 1678 Forward** | **ATGGCGGCACCCAGCATGAAACCCC** |
| **βPIX Reverse** | **TCATAAATTTGTCTCATCCCAAGAAGGATC** |
